# Root and Shoot Biomass Growth of Constructed Floating Wetlands Plants in Saline Environments

**DOI:** 10.3390/ijerph16020275

**Published:** 2019-01-18

**Authors:** Oriana Sanicola, Terry Lucke, Michael Stewart, Katharina Tondera, Christopher Walker

**Affiliations:** 1 Stormwater Research Group, University of the Sunshine Coast, Sunshine Coast, QLD 4558, Australia; tlucke@usc.edu.au (T.L.); mrs017@student.usc.edu.au (M.S.); chrisw@covey.com.au (C.W.); 2 IMT Atlantique, Process Engineering for Environment and Food, Université Bretagne Loire, F-44307 Nantes, France; Katharina.tondera@rwth-aachen.de; 3 Covey Associates Pty Ltd., Maroochydore, QLD 4558, Australia

**Keywords:** constructed floating wetlands, stormwater pollution, plant biomass

## Abstract

Constructed Floating Wetlands (CFWs) are increasingly being used globally in freshwater environments such as urban lakes and ponds to remove pollutants from urban stormwater runoff. However, to date there has been limited research into the use and performance of these systems in saline environments. This study compared the root and shoot biomass growth and nutrient uptake of five different plant species, *Chrysopogon zizanioides*, *Baumea juncea*, *Isolepis nodosa*, *Phragmites australis* and *Sarcocornia quinqueflora*, in three different saltwater treatments over a 12-week period. The aim of the study was to identify which of the plant species may be most suitable for use in CFWs in saline environments. Plant nutrient uptake testing revealed that *Phragmites australis* had the greatest percentage increase (1473–2477%) of Nitrogen mass in the shoots in all treatments. *Sarcocornia quinqueflora* also had impressive Nitrogen mass increase in saltwater showing an increase of 966% (0.208 ± 0.134 g). This suggests that the use of *Phragmites australis* and *Sarcocornia quinqueflora* plants in CFWs installed in saline water bodies, with regular harvesting of the shoot mass, may significantly reduce Nitrogen concentrations in the water. *Isolepis nodosa* had the greatest percentage increase (112% or 0.018 ± 0.020 g) of Phosphorous mass in the shoots in the saltwater treatment. *Baumea juncea* had the greatest percentage increase (315% or 0.026 ± 0.012 g) of Phosphorous mass in the roots in the saltwater treatment. This suggests that the use of *Isolepis nodosa* and *Baumea juncea* plants in CFWs installed in saline water bodies may significantly reduce Phosphorous concentrations in the water if there was a way to harvest both the shoots above and the roots below the CFWs. The study is continuing, and it is anticipated that more information will be available on CFW plants installed in saline environments in the near future.

## 1. Introduction

**Figure 1:**
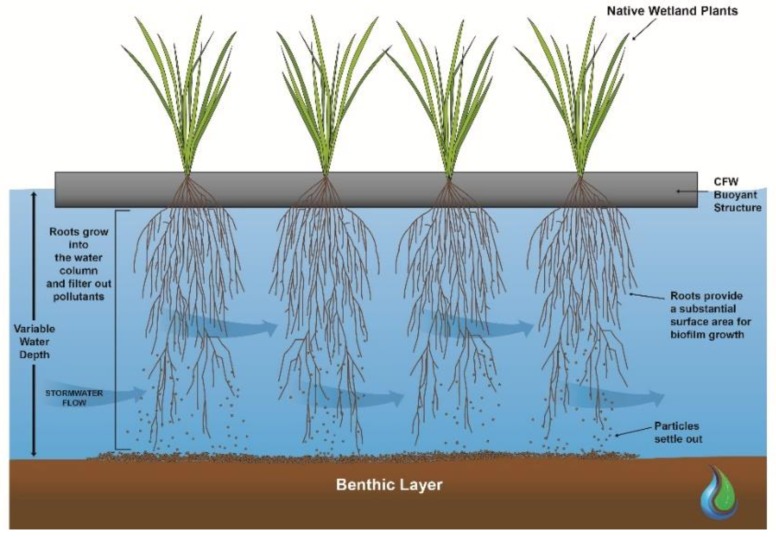
Schematic of Constructed Floating Wetland.

Constructed Floating Wetlands (CFWs) are an innovative stormwater treatment technology, designed to mimic both the structure and function of naturally occurring floating wetlands [1]. CFWs consist of buoyant artificial medium which floats on the water surface and is planted with emergent wetland plants. The plant roots grow through the artificial matrix and into the water column below (Figure 1). The plant roots also adsorb nutrients directly and provide an extensive surface area for the growth of biofilms and sedimentation of suspended particles [2,3,4].

Over the last two decades, CFWs have been used for water quality improvement in various applications, including the treatment of sewage, industrial wastewater, water supply reservoirs and stormwater runoff [2]. The economic benefits of CFWs have been widely reported [3,5,6], including low manufacturing, installation and maintenance costs, and the fact that CFWs generally require no extra land uptake as they can be retrofitted into existing water bodies.

Compared to constructed terrestrial wetlands, CFWs have several advantages in the treatment of stormwater runoff. The buoyancy of the structure allows for adjustment to the varying water depths that are typically seen in event-driven systems [3,6]. Also, the substantial surface area for biofilm growth within the extensive root network improves their nutrient uptake and pollutant removal performance [7]. The microbes within the biofilm contribute to the nutrient removal performance of CFWs [5,8,9].

Therefore, optimizing root development and maximizing the contact area between the root biofilm and the flowing stormwater is an essential design objective for CFWs [7]. The root structure also plays an important part in the pollutant removal performance with a dense network of fibrous roots likely to provide more surface area to treat particulate pollutants than a structure with a non-fibrous root system [10]. Cheng et al. [10] found that Nitrogen removal was significantly higher in fibrous root plant constructed wetlands than rhizomatic root plant wetlands.

There have been numerous studies undertaken in the past to assess the nutrient removal performance of CFWs for stormwater runoff in freshwater environments such as lakes and ponds [1,2,5,6,7,11]. Headley and Tanner [7] reported on different plant species that had been used in CFWs worldwide for water quality enhancement in freshwater environments. However, research into the use of CFWs for the treatment of stormwater runoff in saline environments has been limited to date.

Plant growth is known to be inhibited by high salinity levels in soil [12,13,14], and in water [15,16]. This is commonly referred to as salt stress. Plant species vary greatly in their tolerance to salinity and this is reflected by their growth response [13,17]. Given the importance of the CFW plant root network in nutrient uptake and pollutant removal from stormwater, satisfactory plant root biomass growth is essential to optimize their pollution treatment performance in saline environments.

Other factors to consider in plant selection for CFWs include aesthetics, robustness and endemicity [7]. CFWs for the treatment of stormwater are often located in populated urban areas and it is, therefore, important to design an aesthetically pleasing floating wetland. Selecting plant species that are native to the local area ensures aesthetic integration of the treatment device into the natural environment as well as providing a habitat for local wildlife and maintaining the integrity of the local ecosystem [9]. In particular, floating wetlands installed in urban developments often attract a variety of birdlife. Therefore, selecting robust native plants that can withstand the stresses typically associated with wild birdlife, as well as deterring pest species (e.g., ibis in Australia), may also be a consideration.

A new CFW installation in a saline canal is planned to treat stormwater runoff from a new residential development in south east Queensland in Australia. As there has only been minimal research undertaken on CFWs installed in saline environments to date, there was little information available about which plants would be suitable for use in these conditions. This paper reports on a 12-week mesocosm study that investigated the suitability of five different CFW plant species to treat urban runoff in saline environments. Root and shoot biomass growth was monitored in three different salinity treatments to determine how the different salinity levels affected plant growth. The aim of the study was to identify which of the plant species may be suitable for use in CFWs in saline environments.

## 2. Materials and Methods 

### 2.1. Preliminary Study

To determine a water parameter baseline for replication in the mesocosm trials, the water quality in the saline canal at the intended installation site was monitored for a period of ten weeks prior to commencing the study. A variety of parameters including dissolved oxygen (DO) (% sat), salinity (ppt), pH, electrical conductivity (µS/cm) and temperature (°C) were measured and recorded at two depths, and three locations in the canal. This data was used to inform the water conditions required for this study.

The plant species used in the trial were selected after extensive consultation with local Council arborists and industry experts and tubestock was sourced from a local nursery. The five species recommended for the mesocosm salinity trial were *Chrysopogon zizanioides* (Vetiver), *Baumea juncea* (Twigrush), *Isolepis nodosa* (Knobby club-rush), *Phragmites australis* (Common reed) and *Sarcocornia quinqueflora* (Samphire).

### 2.2. Plant Biomass Study

To assess the effect of salinity on the plant growth, three different water treatments were investigated, namely, saltwater, freshwater and freshwater with increasing salinity levels. Saltwater was sourced from the intended CFW installation site. Freshwater was sourced from an urban lake in another local residential development that had similar environmental and catchment conditions to the intended CFW installation site. The third treatment investigated in the study also used the freshwater from the urban lake. However, the salinity levels in this water increased gradually over the trial period until the salinity levels were similar to those at the intended CFW installation site. This was done to investigate whether the plants displayed better adaptation to a gradual increase in salinity, rather than being planted directly into a high salinity environment.

The study was conducted over a period of 12 weeks as this was considered to be a suitable time frame to allow the plants to grow large enough to be able to undertake the experiments but not so large that they would outgrow their planting containers. Salinity levels were monitored throughout the 12-week trial using a Hanna Instruments 98194 multi-parameter probe and adjusted, if required, using specialized, artificial reef salt. The saltwater treatment tubs were maintained at 30 ppt while the increasing salinity tubs increased by 3 ppt per week until 30 ppt was reached in the final weeks of the study. No salt was added to the freshwater tubs during the study as these were used as a control.

**Figure 2:**
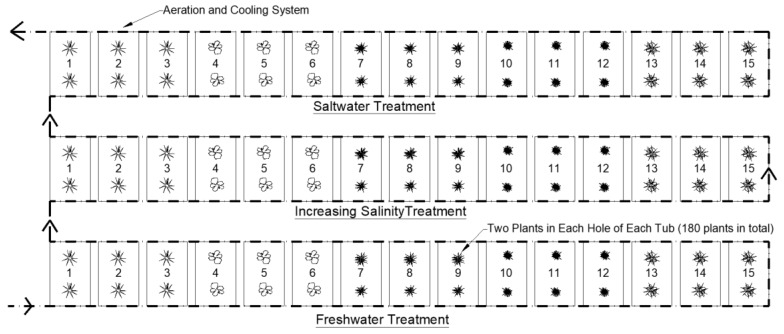
Plant Salinity Evaluation Study Setup.

Three replicate tubs were set up for each of the five study plant species (45 tubs in total). The replicate tubs were arranged in three rows according to the salinity condition being assessed (Figure 2). Commercially available CFW matting was cut to fit the 45 mesocosm tubs with two planting holes per mat. Two plants were planted in each of the two holes per mat for the five different species. This set up was replicated for each of the three water treatments, resulting in a total of 180 individual plants used in the study (Figure 2).

A cooling system was designed for the mesocosm study to keep the water temperature inside the tubs consistent and similar to the baseline determined from the field site monitoring data. Similarly, an aeration system was designed and installed on the tubs to ensure that DO levels remained within the range recorded from the field monitoring (Figure 2).

### 2.3. Plant Measurements 

To quantify the change in shoot and root biomass during the 12 weeks of the study after planting, four representative tubestock plants from each species were analyzed to determine a representative biomass baseline. The four tubestock plants were separated from their potting mix, washed gently in water, and dissected into shoots and roots. The individual shoots and root biomass samples were dried at 70 °C for 7 days and then weighed. The tubestock plant samples were also analyzed to determine the Nitrogen and Phosphorous concentrations in the shoots and roots.

**Figure 3:**
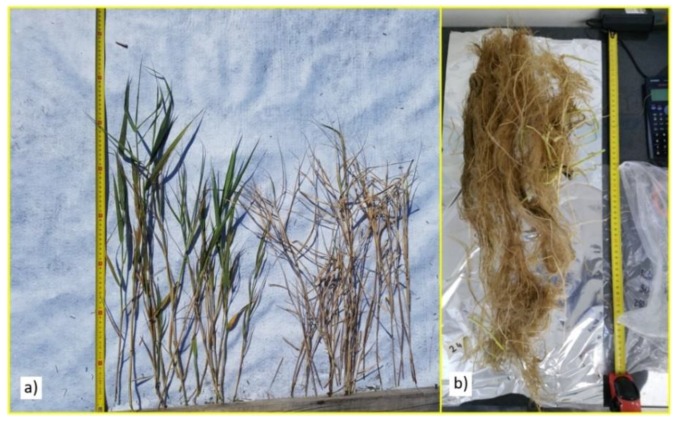
Measurement of Shoots (**a**) and Roots (**b**) of *Phragmites australis* plants after 12 weeks.

All plants were removed from the CFW tubs at the end of the 12-week mesocosm trial and weighed to determine the shoot and root biomass quantities (Figure 3). These were compared with the initial tubestock results to determine the relative change in biomass. The total increase in root and shoot biomass for each plant species at 12 weeks after planting was calculated by subtracting the average initial biomass of the tubestock plants from the average plant biomass at 12 weeks after planting and multiplying this by the total number of plants.

### 2.4. Nutrient Analysis 

To evaluate the changes in nutrient uptake by the different plants between the three water treatments, samples of plant shoots and roots after 12 weeks’ growth were sent to a specialist laboratory and analyzed to determine their Nitrogen and Phosphorous concentrations. These were then compared to the initial shoot and root nutrient values to determine the amount of nutrient in each part of the plant. The total increase in root and shoot nutrient for each plant species at 12 weeks after planting was calculated by subtracting the average initial nutrient mass of the tubestock plants from the average plant nutrient mass at 12 weeks after planting.

## 3. Results

### 3.1. Shoot Growth

**Figure 4:**
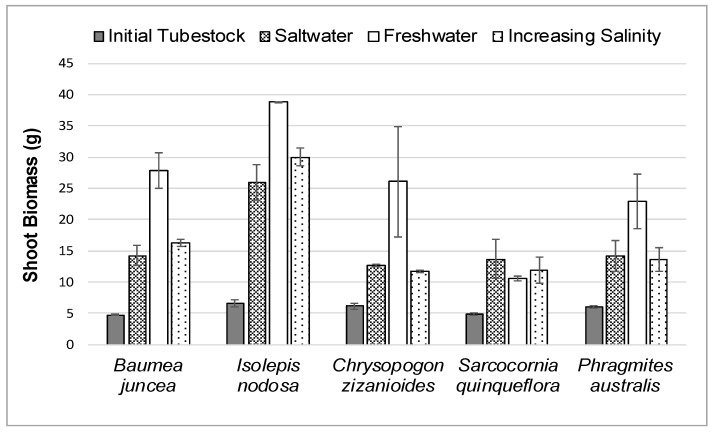
Average Initial and Final Shoot Biomass for Each Species in Each Water Treatment.

The average initial and final shoot biomass for each plant species in the three different salt treatments during the 12-week study is shown in Figure 4.

The average initial and final shoot growth mass for each plant was determined by removing and weighing the representative plant samples as discussed above. These results are shown in Table 1. Shoot growth rate was calculated as a percentage change from the initial tubestock shoot mass at the start of the study until the plants were harvested at the end of the study. These values are shown for each species in brackets in Table 1.

The initial and final shoot nutrient mass for each plant species in the three different salt treatments during the 12-week study is shown in Table 2. The percentage change in shoot nutrient mass for each species is shown in brackets in Table 2.

As shown in Figure 4 and Table 1, the shoot biomass growth of all plant species in salt or increasing saltwater treatments was less than the biomass growth in freshwater except for the *Sarcocornia quinqueflora* plants. The *Baumea juncea* and *Isolepis nodosa* plants appeared to respond better to gradually increasing the salt levels than placing the plants directly into the full salt concentration. However, gradually increasing the salt concentrations had little effect on the shoot growth of *Chrysopogon zizanioides* or *Phragmites australis*.

Table 2 shows that *Phragmites australis* had the greatest percentage increase of Nitrogen mass in the shoots in all treatments (1473–2477%). The *Sarcocornia quinqueflora* plants also had impressive Nitrogen mass increase in saltwater showing an increase of 966% (0.208 ± 0.134 g). *Isolepis nodosa* had the greatest percentage increase (112% or 0.018 ± 0.020 g) of Phosphorous mass in the shoots in the saltwater treatment.

### 3.2. Root Growth

**Figure 5:**
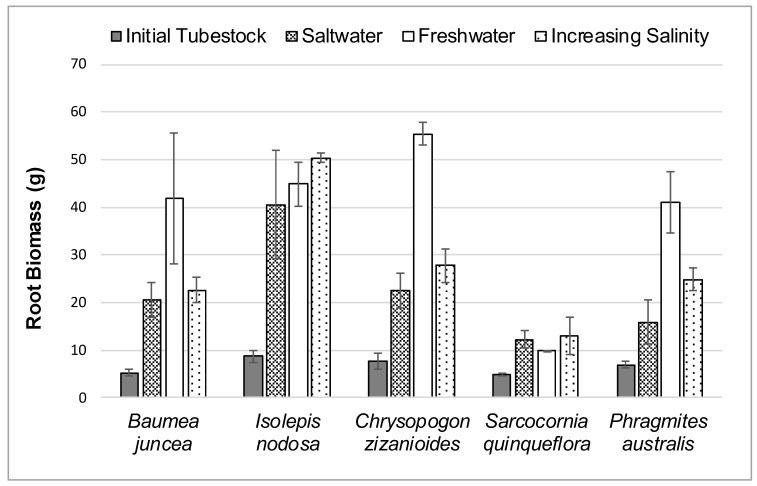
Average Initial and Final Root Biomass for Each Species in Each Water Treatment.

The average initial and final root biomass for each plant species in the three different salt treatments during the 12-week study is shown in Figure 5.

Root growth rate was calculated as a percentage change from the initial tubestock root mass at the start of the study until the plants were harvested at the end of the study. The percentage change in root mass for each species is shown in brackets in Table 3.

Once again, *Sarcocornia quinqueflora* displayed a better root biomass growth rate in saltwater than freshwater. All five species displayed slightly better root biomass growth response rates in the gradually increasing salinity treatment than the full salt concentration.

The initial and final root nutrient mass for each plant species in the three different salt treatments during the 12-week study is shown in Table 4. The percentage change in root nutrient mass is shown in brackets.

Table 4 shows that *Phragmites australis* again had the greatest percentage increase of Nitrogen mass in the roots in all treatments (1,412%–4,484%). While, *Baumea juncea* had the greatest percentage increase (315% or 0.026 ± 0.012 g) of Phosphorous mass in the roots in the saltwater treatment.

## 4. Discussion

Previous research has demonstrated that plant root biomass plays a critical role in the stormwater treatment performance of CFWs [7,9,17,18,19]. Other studies have quantified root biomass growth and shown that satisfactory stormwater pollutant removal performance by CFWs was directly related to root biomass quantities [3,18].

**Figure 6:**
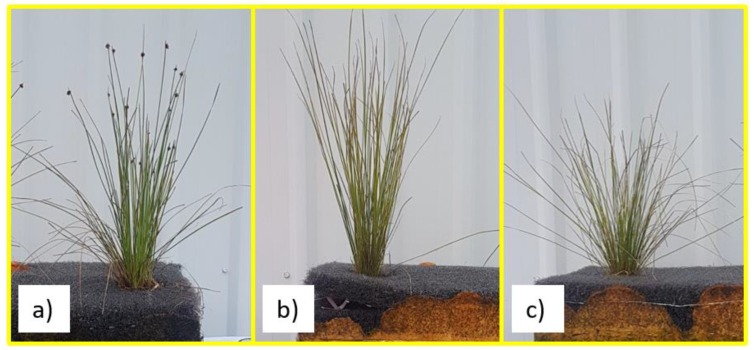
*Isolepis nodosa* Shoots at the End of the 12 Week Study in (**a**) Freshwater Treatment (**b**) Increasing Salinity Treatment and (**c**) Saltwater Treatment.

The results of this study suggest that in terms of root and shoot biomass growth, *Isolepis nodosa* may be the most suitable plant species for use in CFWs installed in saline environments. The *Isolepis nodosa* plants had the greatest increase in shoot biomass in all three water treatments over the 12-week study. Shoots of this species in each of the water treatments can be seen in Figure 6.

While the growth rate for root biomass was not as high as some of the other plants in freshwater, it was the greatest of all five plants in the increasing salt (585%), and the full salt treatments (471%). This root biomass growth is essential for pollutant removal from stormwater runoff. This species is also native to Australia and is robust enough to withstand potential stressors caused by local wildlife.

**Figure 7:**
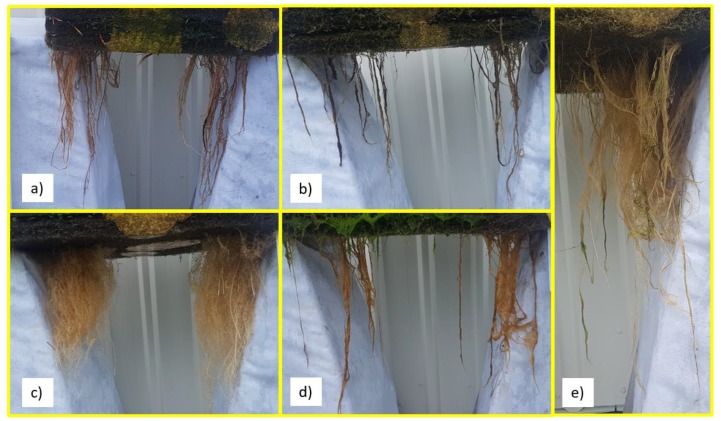
Root Systems of (**a**) *Baumea juncea*; (**b**) *Isolepis nodosa*; (**c**) *Chrysopogon zizanioides*; (**d**) *Sarcocornia quinqueflora* and (**e**) *Phragmites australis* plants.

It has been suggested [10] that root structure plays a role in the pollutant removal performance of wetland plants, with fibrous root systems providing more surface area to trap particulate matter and demonstrating higher Nitrogen removal. The root systems for the five different plant species in the treatments which resulted in the best root biomass growth throughout the study can be seen in Figure 7.

Figure 7 shows the plants with the densest and more fibrous network of roots are *Chrysopogon zizanoides* (Figure 7c) and *Phragmites australis* (Figure 7e). However, despite the apparently large root system (Figure 7e), *Phragmites australis* has a rhizomatic root system with a much smaller number of fine secondary roots which may be less advantageous in the trapping of fine particles. Also, despite a large increase in root biomass over the 12-week study, the *Baumea juncea* (Figure 7a) and *Isolepis nodosa* (Figure 7b) plants do not appear to have the dense network of fibrous roots associated with higher pollutant removal rates [10].

Despite its relatively low shoot biomass growth and less dense root network, Table 2 and Table 4 show that *Phragmites australis* had the greatest percentage increase of Nitrogen mass in both the shoots and roots, in all treatments. Isolepis nodosa had the greatest percentage increase (112%) of Phosphorous mass in the shoots in the saltwater treatment, while, *Baumea juncea* had the greatest percentage increase (315% or 0.026 ± 0.012 g) of Phosphorous mass in the roots in the saltwater treatment.

When considering the root structure, *Chrysopogon zizanoides* (Figure 7c) displayed a dense network of fibrous roots with a very high number of secondary lateral roots. It also displayed a significant increase in root biomass in the increasing salinity treatment over the 12-week study period. This combination of root structure and biomass suggested that this species may also have been a suitable choice for use in CFWs in saline environments. However, the results in Table 2 and Table 4 show that the nutrient uptake of these plants in saltwater was not encouraging.

For aesthetic reasons it may often be appropriate to include a variety of plants in a floating wetland design, including groundcover plants as well as grasses and sedges. Plants that attract native wildlife may also play a role in the plant selection process. For example, *Sarcocornia quinqueflora* is a groundcover plant that would offer some variety to the visual aspect of the CFWs, and, given that it exhibited better root growth in saltwater than in freshwater, could be considered for use in saline environments. However, further studies are recommended to assess whether this species shows improved root growth over a longer timeframe.

## 5. Conclusions

This paper reports on a 12-week mesocosm study that investigated the suitability of five different CFW plant species to treat urban runoff in saline environments. Root and shoot biomass growth was monitored in three different salinity treatments to determine how the different salinity levels affected plant growth. The five species recommended for the mesocosm salinity trial were *Chrysopogon zizanioides* (Vetiver), *Baumea juncea* (Twigrush), *Isolepis nodosa* (Knobby club-rush), *Phragmites australis* (Common reed) and *Sarcocornia quinqueflora* (Samphire).

The study results showed that the shoot biomass growth of the *Sarcocornia quinqueflora* plants in saltwater (30 ppt) or increasing salt water (increasing freshwater by 3 ppt per week up to 30 ppt) was greater than in freshwater. The shoot biomass growth of all other plants was less in saltwater or increasing saltwater than in freshwater. *Sarcocornia quinqueflora* also displayed a better root biomass growth rate in saltwater than freshwater. All five species displayed slightly better root biomass growth response rates in the gradually increasing salinity treatment than the full salt concentration. These results demonstrate that Sarcocornia *quinqueflora* is a hardy plant this may be suitable for use in CFWs in saline environments.

Plant nutrient uptake testing revealed that *Phragmites australis* had the greatest percentage increase (1473–2477%) of Nitrogen mass in the shoots in all treatments. The *Sarcocornia quinqueflora* plants also had impressive Nitrogen mass increase in salt water showing an increase of 966% (0.208 ± 0.134 g). This suggests that the use of *Phragmites australis* and *Sarcocornia quinqueflora* plants in CFWs installed in saline water bodies, with regular harvesting of the shoot mass, may significantly reduce Nitrogen concentrations in the water.

*Isolepis nodosa* had the greatest percentage increase (112% or 0.018 ± 0.020 g) of Phosphorous mass in the shoots in the saltwater treatment. *Phragmites australis* also had the greatest percentage increase of Nitrogen mass (1,412–4,484%) in the roots in all treatments, while, *Baumea juncea* had the greatest percentage increase of Phosphorous mass in the roots in the saltwater treatment (315% or 0.026 ± 0.012 g). This suggests that the use of *Isolepis nodosa* and *Baumea juncea* plants in CFWs installed in saline water bodies may significantly reduce Phosphorous concentrations in the water if there was a way to harvest the roots underneath the CFWs.

The study is continuing, and it is anticipated that more information will be available on CFW plants installed in saline environments in the near future. In particular, it is anticipated that the relationship between pollutant removal performance and the root structure and biomass will be clearly identified.

## Figures and Tables

**Table 1:** Initial Tubestock Shoot Mass and Final Plant Shoot Mass for Each Species in Each Water Treatment (Percentage Change in Shoot Mass Shown in Brackets).

Plant Species	Average Shoot Mass (g)			
	Initial	Saltwater	Freshwater	Increasing Salinity
*Baumea juncea*	4.72 ± 0.12	14.24 ± 1.56 (302%)	27.81 ± 2.84 (589%)	16.26 ± 0.59 (344%)
*Isolepis nodosa*	6.54 ± 0.60	26.02 ± 2.85 (398%)	39.96 ± 0.01 (596%)	29.99 ± 1.40 (459%)
*Chrysopogon zizanioides*	6.10 ± 0.41	12.71 ± 0.21 (208%)	26.08 ± 8.78 (427%)	11.68 ± 0.26 (192%)
*Sarcocornia quinqueflora*	4.91 ± 0.17	13.65 ± 3.18 (278%)	10.55 ± 0.36 (215%)	11.90 ± 2.13 (242%)
*Phragmites australis*	5.98 ± 0.25	14.17 ± 2.56 (237%)	22.94 ± 4.31 (384%)	13.63 ± 1.87 (228%)

**Table 2:** Initial Tubestock Shoot Nutrient Mass and Final Shoot Nutrient Mass for Each Species in Each Water Treatment (Percentage Change in Shoot Nutrient Mass Shown in Brackets).

Plant Species	Average Shoot Nitrogen Mass (g)				Average Shoot Phosphorus Mass (g)			
	Initial	Saltwater	Freshwater	Increasing Salinity	Initial	Saltwater	Freshwater	Increasing Salinity
*Baumea juncea*	0.022 ± 0.002	0.137 ± 0.045 (519%)	0.222 ± 0.054 (905%)	0.146 ± 0.016 (563%)	0.007 ± 0.003	0.012 ± 0.007 (66%)	0.031 ± 0.005 (341%)	0.018 ± 0.006 (158%)
*Isolepis nodosa*	0.083 ± 0.013	0.171 ± 0.043 (107%)	0.292 ± 0.128 (253%)	0.079 ± 0.054 (−5%)	0.016 ± 0.007	0.034 ± 0.013 (112%)	0.060 ± 0.033 (273%)	0.039 ± 0.007 (142%)
*Chrysopogon zizanioides*	0.049 ± 0.008	0.083 ± 0.019 (71%)	0.168 ± 0.097 (245%)	0.079 ± 0.011 (61%)	0.012 ± 0.002	0.011 ± 0.008 (-10%)	0.062 ± 0.023 (425%)	0.009 ± 0.006 (−20%)
*Sarcocornia quinqueflora*	0.022 ± 0.008	0.229 ± 0.126 (966%)	0.132 ± 0.024 (798%)	0.194 ± 0.054 (513%)	0.013 ± 0.002	0.022 ± 0.007 (68%)	0.016 ± 0.004 (23%)	0.022 ± 0.009 (68%)
*Phragmites australis*	0.012 ± 0.001	0.185 ± 0.080 (1473%)	0.304 ± 0.089 (1682%)	0.210 ± 0.045 (2477%)	0.016 ± 0.013	0.017 ± 0.008 (1%)	0.040 ± 0.023 (145%)	0.021 ± 0.006 (30%)

**Table 3:** Initial Tubestock Root Mass and Final Plant Root Mass for Each Species in Each Water Treatment (Percentage Change in Root Mass Shown in Brackets).

Plant Species	Average Root Mass (g)			
	Initial	Saltwater	Freshwater	Increasing Salinity
*Baumea juncea*	5.22 ± 0.63	20.47 ± 3.59 (392%)	41.82 ± 13.78 (801%)	22.53 ± 2.65 (432%)
*Isolepis nodosa*	8.61 ± 1.38	40.55 ± 11.45 (471%)	44.78 ± 4.71 (520%)	50.36 ± (585%)
*Chrysopogon zizanioides*	7.69 ± 1.63	22.47 ± 3.73 (292%)	55.33 ± 2.39 (720%)	27.72 ± 3.51 (360%)
*Sarcocornia quinqueflora*	4.79 ± 0.21	12.21 ± 1.89 (255%)	9.75 ± 0.24 (204%)	12.92 ± 3.97 (270%)
*Phragmites australis*	6.92 ± 0.66	15.87 ± 4.67 (229%)	41.02 ± 6.41 (593%)	24.86 ± 2.25 (359%)

**Table 4:** Initial Tubestock Root Nutrient Mass and Final Root Nutrient Mass for Each Species in Each Water Treatment (Percentage Change in Root Nutrient Mass Shown in Brackets).

Plant Species	Average Root Nitrogen Mass (g)				Average Root Phosphorous Mass (g)			
	Initial	Saltwater	Freshwater	Increasing Salinity	Initial	Saltwater	Freshwater	Increasing Salinity
*Baumea juncea*	0.027 ± 0.005	0.102 ± 0.022 (282%)	0.156 ± 0.063 (482%)	0.188 ± 0.058 (603%)	0.008 ± 0.006	0.034 ± 0.007 (315%)	0.062 ± 0.036 (651%)	0.029 ± 0.011 (250%)
*Isolepis nodosa*	0.087 ± 0.015	0.157 ± 0.119 (81%)	0.251 ± 0.055 (190%)	0.250 ± 0.118 (188%)	0.019 ± 0.008	0.055 ± 0.031 (189%)	0.090 ± 0.031 (371%)	0.055 ± 0.015 (190%)
*Chrysopogon zizanioides*	0.040 ± 0.023	0.080 ± 0.027 (102%)	0.288 ± 0.066 (625%)	0.086 ± 0.018 (116%)	0.014 ± 0.005	0.013 ± 0.004 (−6%)	0.060 ± 0.030 (325%)	0.016 ± 0.003 (10%)
*Sarcocornia quinqueflora*	0.029 ± 0.004	0.108 ± 0.027 (273%)	0.107 ± 0.017 (268%)	0.112 ± 0.037 (286%)	0.007 ± 0.003	0.016 ± 0.013 (150%)	0.020 ± 0.006 (195%)	0.017 ± 0.014 (164%)
*Phragmites australis*	0.006 ± 0.001	0.090 ± 0.067 (1,412%)	0.273 ± 0.087 (4,484%)	0.187 ± 0.074 (3,050%)	0.006 ± 0.001	0.023 ± 0.013 (304%)	0.094 ± 0.052 (1516%)	0.034 ± 0.010 (482%)
